# Application of Next Generation Sequencing for personalized medicine for sudden cardiac death

**DOI:** 10.3389/fgene.2015.00055

**Published:** 2015-03-02

**Authors:** Elena Morini, Federica Sangiuolo, Daniela Caporossi, Giuseppe Novelli, Francesca Amati

**Affiliations:** ^1^Department of Movement, Human and Health Sciences, University of Rome Foro ItalicoRome, Italy; ^2^Department of Biomedicine and Prevention, University of RomeTor VergataRome, Italy

**Keywords:** cardiomyopathies, channelopathies, sudden cardiac death, Next-Generation Sequencing, personalized medicine

## Abstract

Sudden cardiac death (SCD) is a serious public health problem. In the United States, more than 300,000 people are affected by SCD every year. Significantly, sudden deaths represent 20% of the total mortality and 50% of cardiovascular mortality in Western countries. In addition, SCD constitutes one of the most important unsolved challenges in the practice of forensic pathology because of the failure to determine the exact cause of sudden death. In young individuals, SCD is frequently caused by cardiomyopathies and channelopathies, that have generally an autosomal dominant pattern of inheritance. The impact of genetics and genetic testing on the clinical management of these diseases is unquestioned. In particular, genetic tests are an important tool for identifying pre-symptomatic individuals carrying genetic variant that predisposes them to SCD. High-throughput sequencing technologies offer novel opportunities to deeper investigate the genetic background underlying these fatal diseases and to early identify individuals at risk for SCD. In this review, we provide an overview of the development of Next-Generation Sequencing (NGS) technologies and of guidelines useful to design an efficient sequencing protocol and to perform an accurate data analysis. We suggest a flow chart to follow for the set up of a genetic screening protocol for the prevention of cardiac pathologies, in particular SCD events, in young athletes.

## INTRODUCTION

DNA sequencing technologies have a broad range of applications in different fields, including molecular cloning and identification of pathogenic genes as well as comparative and evolutionary studies. During the past 30 years, Sanger sequencing has been adopted as the primary technology in the “first generation” of sequencers and has been used in the sequencing of the first human genome (Human Genome Project; Liu et al., 2012). At the beginning of the Human Genome Project, the cost estimate for this “enterprise” was $2.7 billion. In 2005, with the introduction of Next-Generation Sequencing (NGS), the identical procedure was performed for approximately $1.5 million (Wheeler et al., 2008; Liu et al., 2012). A further decrease in the costs of sequencing was occurred after 2005, and the NGS technology will become widely accessible.

In recent years, NGS has been applied to comprehensively study several Mendelian monogenic disorders as well as complex diseases including cancer (Navin and Hicks, 2011) and cardiac diseases (Fokstuen et al., 2014). For these multifactorial diseases, a very important advantage of NGS is the possibility of testing many genes in a relatively short time and with low cost.

Sudden cardiac death (SCD), commonly defined as a natural death from unexplained cardiac causes, is an important social and medical issue (Brion et al., 2010). Interestingly, young athletes are among the categories mostly affected by SCD. In fact, adrenergic stress during competitive sports is a commonly accepted trigger for arrhythmias and SCD, in the presence of an underlying inherited cardiac disease such as cardiomyopathy, primary arrhythmia syndrome or vascular diseases (Corrado et al., 2005). So, great attention has been focused on a molecular analysis of cardiac channelopathies and cardiomyopathies that would allow an early diagnosis and prevention of SCD in a significant percentage of young individuals (Brion et al., 2010).

## NGS PROTOCOL DESIGN

To establish a NGS diagnostic protocol, we must first perform a validation phase to verify that all of the mutations previously found in a group of individuals analyzed with Sanger sequencing are also detectable with the new sequencing technique. After this phase, we could continue with an application phase performed on individuals who were not previously analyzed.

Novel variants identified by NGS must be confirmed by Sanger sequencing to evaluate the reproducibility of the NGS approach. Reproducibility, in a NGS protocol, is intended as confirmation of the results using a traditional Sanger method. If a specific variant revealed by NGS sequencing is not found with Sanger sequencing, it is not accepted.

An important step of the entire NGS protocol is DNA isolation. This step in fact, requires the use of a uniform method to guarantee an equal and standardized quality of genomic DNA. Once the DNA is obtained, the next step is to generate a library by pooling different DNA samples. Each individual sample, inside the DNA pool, must be identifiable during the data analysis after ligation with a barcode (Dorn et al., 2013). The use of the barcode is one of the strengths of the NGS protocol; in fact, a barcode allows the analysis of more patients in the same run cycle, yielding lower analysis costs, and improving clinical response times. The ligation of DNA with barcodes is a very critical step, and great attention is devoted to not exchanging the samples to avoid erroneous results.

Next-Generation Sequencing technologies are suitable for a wide range of study designs but there are some important points to consider: the number of individuals to study, the number of genes to be analyzed and the choice of the sequencing platform. However, for small cohorts, the significance of a NGS study increases if the cohort consists of a homogenous group of individuals (Arad et al., 2005). Another important factor is the number of genes to analyze, particularly for multifactorial pathologies caused by variations in more than one gene. The simultaneous study of a large number of genes makes it possible to underline all of the mutations present in one patient and to analyze the possible correlation between these variants and the pathology. This approach is very expensive and requires long analysis times, which comprise critical points for a diagnostic genetic test. For a molecular diagnosis, we consider useful to focus on a limited and selected group of genes considered to be associated with the pathology under study (Amati and Morini, unpublished data).

In recent years, several platforms for NGS have been developed; their main characteristics are summarized in **Table 1** (modified from Liu et al., 2012; Li et al., 2014).

**Table 1:** The main characteristics of the most common Next-Generation Sequencing (NGS) platforms (modified from Liu et al., 2012; Li et al., 2014).

Sequencers	454 GS FLX (Roche)	HiSeq 2000 (Illumina)	SOLiDv4 (Applied Biosystems)	Ion torrent (Life Technologies)
Methods	Pyrosequencing	Sequencing by synthesis	Sequencing by ligation	Ion semiconductor
Read length	700 bp	50–250 bp	35–50 bp	400 bp
Accuracy*	*Q* > 30	20 < *Q* > 30	*Q* > 30	Q20
Reads per run	1 million	Up to 3 billion	1.2–1.4 billion	Up to 80 million
Time per run	24 h	1–10 days	1–2 weeks	2 h
Cost per 1 million bases	$10	$0.05 to $0.15	$0.13	$1
Advantages	Read length	High throughput	Low cost per base	Less expensive equipment
	Fast		Accuracy	Fast
Disadvantages	Runs expensive	Expensive	Slower method	Homopolymer errors
	Homopolymer errors	High concentrations of DNA	Palindromic sequences errors	
	Low throughput	Short reads	Short read	

*The values of accuracy have been converted in a *Q* score value (Ewing and Green, 1998) and refer to the optimal experimental conditions for each NGS platform. *Q* score is the measure of base calling accuracy (Ewing and Green, 1998). Low *Q* values (Q10) can lead to increase false-positive variant calls.

## NGS GUIDELINES FOR DIAGNOSTIC TESTS

A diagnostic test is a medical test performed to aid in the diagnosis or detection of a disease. A genetic diagnostic test must be very specific and accurate to explain the phenotype of a patient or to indicate the risk of developing a specific disease. A diagnostic test should be carried out by specialized laboratories that produce results in agreement with the quality and competence standard for medical laboratories (ISO 15189 or comparable). In general, a genetic test is performed on a biological sample (commonly a blood sample) of a subject (patient or proband) and the gene/s of interest are sequenced to search mutations. Before sample collection, it is mandatory to obtain informed consent from the patient. Informed consent is defined as a legal document to ensure that the patient is aware of all the potential risks and costs involved in a treatment or procedure. Moreover, an informed consent informs the patient regarding the nature of the treatment, possible alternative treatments and potential risks and benefits of the treatment.

As NGS has been developed as a tool for research applications, it shows an enormous potential application in genetic diagnosis, such as to promote genetic tests for hereditary and congenital disorders (Sikkema-Raddatz et al., 2013). There are different categories of genetic testing based on NGS technologies, as follows: whole exome sequencing (WES), whole genome sequencing (WGS), and Targeted or Panel NGS testing. To select one of these approaches, the finality, sensitivity and probability of finding unknown variants or variants with an uncertain significance as well as the data storage must be considered.

For the development of a diagnostic NGS test, design and validation are essential steps. Design indicates selecting the genes to add to the NGS panel; this step is important because it is not useful to select genes without clear evidence of a disease association (Rehm et al., 2013). It is important to create the so-called “core disease gene lists” that include all of the genes that definitely contribute to the disease. Test validation is a fundamental phase aimed to verify that some parameters, such as the sequencing quality, sensitivity, and reproducibility, are close to the referring parameters (Rehm et al., 2013).

The NGS data analysis produces a complete report that includes the following files: FASTQ (base calls of all the reads produced and the quality score of each base); BAM (the alignment of the reads over the reference genome); Variant Call File (VCF; the chromosomal position, name, and reference genome of each variant).

The amount of data found using a NGS protocol is broad and it is very important to analyze and classify the variants properly and to compare them with the international databases of identified and validated variants (i.e., the OMIM^1^ or the HGMD^2^). The main criteria for the identification of a nucleotide variant are a read coverage (the bases are aligned to a specific nucleotide position) of ≥30-fold and a read percentage (the fraction of bases that differ from the reference sequence) of ≥20 (Voelkerding et al., 2010). In general, a nucleotide variant with an allele frequency <0.2 should be considered as a homozygous reference (WT, wild type); allele frequencies between 0.2 and 0.8 as heterozygous; variants with allele frequencies >0.8 must be considered a homozygous mutation (MUT). Variant annotation is a crucial step in the analysis of NGS data. Variant analysis could be performed using several on-line programs; one of the most commonly used programs is ANNOVAR^3^. However, a manual review of reads through a specific browser (i.e., the IGV Browser of the Broad Institute; Robinson et al., 2011; Thorvaldsdóttir et al., 2013) is required to check the alignment quality and homogeneous coverage of the region in question. In fact, regions with repeated sequences, insertions and deletions might be underestimated in an automatic report obtained by on-line tools. A precise description of each variant is very important, according to the recommendation of the Human Genome Variation Society (HGVS), and inclusion of the genomic coordinates (chr11: g 19207841) for each variant is highly recommended. For information on whether a novel variant is common, it is important to compare the variant with the single nucleotide polymorphism (SNP) Database^4^. If a nucleotide variant is not reported in the main mutation databases (i.e., the OMIM or HGMD), it might be *in silico* analyzed using the on-line tool, PolyPhen-2^5^. This software predicts the possible effect of an amino acid substitution on the structure and function of a human protein using straightforward physical and comparative considerations. In addition, it is possible to verify the conservation of the amino acid variant among the species using the ClustalW2 website^6^. After these *in silico* evaluations, it is worthwhile to study the possible functional effect of the nucleotide variant.

Variants are classified in five categories: pathogenic, likely pathogenic, unclassified (UVs), likely benign and benign variants. A problematic aspect of a NGS diagnostic test is the possibility of detecting unsolicited and secondary findings (so called incidental findings, IF). It is necessary for laboratories to have a defined statement for including or not including IF in the final report, in accordance with ethical committees. Reports on the NGS results should follow the general principles of clinical genetic reporting (Claustres et al., 2014) and be in agreement with international diagnostic standards, ISO 15189, and with professional guidelines such as those issued by the Clinical Molecular Genetics Society (CMGS) in the UK (Treacy and Robinson, 2013), by the Human Genetics Society of Australasia^7^ and by the Swiss Society of Medical Genetics^8^ .

A report must be clear, succinct and interpretable by a non-expert; however, it must contain sufficient data for understanding which genes were investigated and which NGS protocol was used (i.e., information regarding the kits, the NGS platforms or the pipeline versions). A report should summarize the patient’s clinical history and diagnosis as well as the variants identified. It is essential that the report mention whether the pathogenic variants have been confirmed by another independent method (Sanger). All the limitations of the diagnostics test should be listed in the report. Among these limitations are repetitive sequences, pseudogenes, homologous regions, the GC content, transversions, or inversions that could not be detected and/or are disregarded.

For polygenic or multifactorial pathologies in which more genes are involved, such as cardiomyopathies, NGS analysis could produce a significant increase in the diagnostic yield. A “diagnostic yield” is defined as the probability that a disease-causing variant is identified and a molecular diagnosis could be made, with the likelihood that a test would provide the information needed to establish a diagnosis (Weiss et al., 2013). The “diagnostic yield” might be a good instrument for measuring the efficiency of a genetic test.

## NGS FOR DIAGNOSIS OF HEREDITARY CARDIAC DEFECTS

Before starting a NGS study, it is important to define the sequencing approach. It is possible to analyze WES and/or WGS. WGS allows observation of all the nucleotide variations in the coding and in non-coding regions while WES identifies variations in all of the coding regions and appears to be a promising option for the study of rare inherited disorders caused by mutations in protein-coding sequences (Dorn et al., 2013). Even if the cost per base is the same, it clear that the cost of WGS is higher than of WES because the amount of bases to sequence is higher; the high costs of the WGS procedure often limit its application.

If the genomic region and/or the gene responsible for one disease are known, target resequencing is an optimal alternative. Targeted NGS tests offer the best solution for the genetic screening of complex diseases, including inherited cardiac diseases, in which different mutations in a large number of genes are involved.

Next-Generation Sequencing platforms have peculiar characteristics (**Table 1**). An important feature is accuracy that refers to the correctness of the sequences, that is base quality, mapping quality, duplicated reads, GC content, strand bias, presence of repetitive sequences and existence of pseudogenes. A measure of accuracy is the miscalling of bases (mismatches) that can cause substantial trouble for example for the identification of SNPs. A recent sequencing study on several NGS platforms shows that the SOLiD and the Roche 454 platforms provide high-quality sequences, while the calling accuracy of Illumina and Ion PGM sequencing platforms are slightly lower (Li et al., 2014). The choice between several platforms depends on the aim and the type of study being performed.

Different NGS platforms have been used for the molecular genetic screening of inherited cardiac conditions. Li et al. (2013) use two different platforms, MiSeq (Illumina, San Diego, CA, USA), and Ion Torrent PGM (Life Technologies Ldt, Paisley, UK), to investigate six genes (*KCNQ1, KCNH2, SCN5A, KCNE1, KCNE2, RYR2*) associated with inherited arrhythmia syndromes. Ion PGM is less expensive and fast, whereas MiSeq has a lower run time and higher capacity. In any case, both platforms are valid instruments for a molecular diagnosis of inherited cardiac diseases because they are faster, less expensive and more comprehensive than traditional genetic diagnostic tests.

A recent diagnostic protocol based on Ion Torrent PGM was performed by Millat et al. (2014). A cohort of patients, previously studied by HRM/sequencing, was used to validate an Ion chip panel designed to identify mutations in the following genes: *MYH7, MYBPC3, MYL2, LMNA, SCN5A, TNNT2, TNNI3* and *TPM1*. All of the substitutions previously found in the *MYBPC3* gene were confirmed whereas some indels were not identified by NGS analysis. Most of these indels were located in the homopolymer regions (long poly A/T regions could cause problems in DNA sequencing reactions because of “slippage”); this limit is present in other NGS platforms (such as 454Roche) but the accuracy of these NGS platforms doesn’t really suffer from it (**Table 1**).

Meder et al. (2011), used another NGS approach (TargetSeq) and SOLiD platform (Applied Biosystem, Carlsbad, CA, USA) for the analysis of 10 patients with cardiomyopathies (HCM and DCM). They found mutations in the *MYBPC3*, *MYH7* and *LMNA* genes as well as novel variants. To confirm the specificity of their results, they selected 50 of these new variants and confirmed their existence by the Sanger method.

All these data demonstrated that the application of NGS, which allowed investigators to analyze more patients and more genes in shorter amounts of time, has been successful for the molecular diagnosis of hereditary cardiac defects.

## NGS: A PROMISING TOOL FOR THE DIAGNOSIS OF SCD

The published data regarding the NGS method and diagnosis of SCD are limited; results from previous studies are very interesting, and they emphasize that NGS could be a useful investigative method for clarifying the role of pathogenetic variants in the risk of developing SCD (Campuzano et al., 2014; Hertz et al., 2014). A recent paper estimated the frequency of pathogenetic variants in 34 genes related to SCD in forensic patients and in patients with channelopathies; the conclusion of this work is that NGS could be considered an important tool for genetic screening of SCD (Hertz et al., 2014). Another study was conducted to validate the SOLiD System with the aim to analyze 28 genes known to be associated with inherited arrhythmogenic disorders and, therefore, with SCD (Brion et al., 2014).

These studies showed that NGS allows accurate detection of many variants using a rapid and less expensive method and it could be a promising diagnostic tool for cardiomyopathies, channelopathies and SCD.

## NGS TEST FOR IDENTIFICATION OF ATHLETES AT RISK OF SCD

**FIGURE 1:**
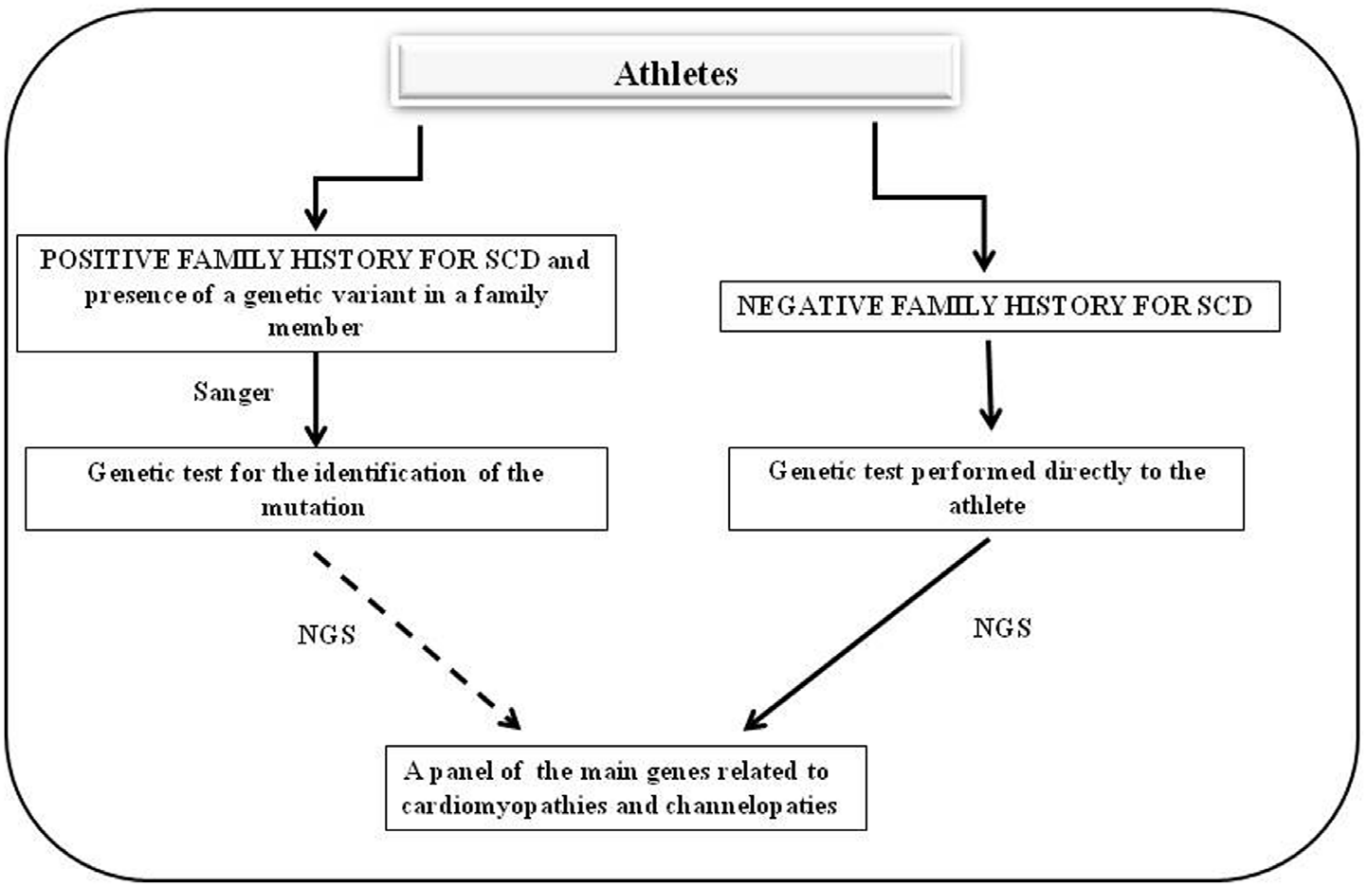
**Flow chart of genetic screening for athletes with suspected cardiac diseases.** The left side shows the diagnostic protocol to follow in cases in which there is a positive family history for cardiac hereditary disease whereas the right side presents the cases in which there is no family history of cardiac hereditary disease and the athlete shows cardiac abnormalities on an ECG or echocardiography.

A close collaboration between clinicians and geneticists is very important for a comprehensive genetic analysis. This collaboration includes the mutual exchange of clinical and laboratory information to explicate the complexity of the process of genomic variant interpretation. Family history and patient symptoms could direct the investigators in the selection of the study, which would reduce the waiting time for patients. Genetic testing must be developed in medical laboratories in which genetic counseling and medical management of patients and relatives with inherited pathologies could be performed. These basic principles are applicable to genetic screening in asymptomatic patients including high-level athletes at risk of SCD. In this case, two situations are possible (**Figure 1**).

The first situation is found in cases in which there is a family history of cardiac hereditary disease and in which one mutation is identified in the athlete’s family. In these cases, genetic testing could be proposed to the athlete to search for the specific mutation identified in his/her family. If this mutation is identified in the athlete it is possible to stop here. However, if there is the suspect that more than one gene may be involved, it is necessary to use a massive approach by NGS. The second situation, which is the most common, is found in cases in which there is no family history of hereditary cardiac disease but the athlete shows cardiac abnormalities on an ECG or echocardiography. In these cases, a genetic test could be proposed directly to the athlete; the test must analyze the main genes associated with hereditary cardiac defects using an NGS approach.

In both situations, it is important that the molecular laboratory for the genetic testing be equipped to develop functional studies to verify all the variants identified in order to evaluate the pathological effect of these ones.

The test results could be used for an individual’s medical treatment and to identify other at-risk family members.

## CONCLUSION

Next-Generation Sequencing offers novel opportunities to study and diagnose complex and multifactorial diseases; however, the economic costs of these analyses remain too high to conduct NGS on the general population. In the future, a reduction of NGS costs would ensure that this diagnostic procedure is universally accessible. An essential direction for the study of cardiac/cardiovascular pathology would be to use additional approaches, including proteomics and systems biology, to enable researchers to filter and combine genetic data for a more complete overview of these complex diseases. Until then, strong integration and collaboration between clinicians and molecular biologists/geneticists are necessary for personalized, careful screening and counseling for individuals at risk for SCD.

## Conflict of Interest Statement

The authors declare that the research was conducted in the absence of any commercial or financial relationships that could be construed as a potential conflict of interest.

## Footnotes

1 http://www.ncbi.nlm.nih.gov/omim

2 http://www.hgmd.cf.ac.uk/ac/index.php

3 http://www.openbioinformatics.org/annovar/

4 http://www.ncbi.nlm.nih.gov/snp

5 http://genetics.bwh.harvard.edu/pph2/

6 http://www.ebi.ac.uk/Tools/msa/clustalw2/

7 https://www.hgsa.org.au/hgsanews/guidelines-for-implementation-of-massively-parallel-sequencing

8 href="http://www.sgmg.ch/user_files/images/SGMG_Reporting_Guidelines.pdf
